# Occupational exposure, knowledge of standard precautions and behavior among health workers in Southern Nigeria

**DOI:** 10.1186/1471-2334-14-S2-P8

**Published:** 2014-05-23

**Authors:** Samuel Ereh

**Affiliations:** 1 Eku Baptist Hospital, Department of Family Medicine, Eku, Nigeria

## Background

Developing countries account for the highest prevalence of HIV-infected patients in the world and also record the highest needlestick injuries. The objective of this study was to assess the exposure, knowledge and practices of standard precautions among Health care workers (HCWs) in Southern Nigeria.

## Methods

We surveyed 212 HCWs in 4 state-owned hospitals and 25 local government primary health centers. Structured questionnaires were utilized. Descriptive statistics and multivariate analysis using logistic regression were done.

## Results

57.7% of respondents were aware of the term Standard Precautions (SPs) overall. Doctors (94.4%) and nurses (84.2%) had the highest levels of awareness compared to 3.0% of health assistants. There was a significant relationship between the respondents' level of education and a correct knowledge of SPs. (Fisher's Exact= 125.745, df = 4, p = 0.000) and duration of employment was not significantly associated with the awareness of SPs. (x2 = 5.676, df =3, p =0.128). Life time risks of needle stick (39.8%; 95% CI 36.4-44.6%) and sharps injuries (25.7%; 95% CI 21.8-29.6%) were high. 77 .8% washed their hands at the close of work. Over half (56.6%) always used gloves and two-thirds (62.3%) had never worn goggles during contact with blood and body fluids. Only 46.2% of HCWs reported washing their hands before each patient contact. Overall compliance with non-recapping of used needles was generally poor (14.6%) and about half (48.1%) of the respondents recapped used needles before disposal. The one year prevalence of needle stick and sharps injury were 29.3% (95% CI 26.7-32.8%) and 22.9% (95% CI 19.8-25.2%) respectively.

## Conclusions

The poor knowledge and non-compliance with SPs among the HCWs placed them at significant health risks. Adequate supply of protective equipment with regular training programs should therefore be put in place to promote appropriate use by the HCWs at all times.

